# The effects of precarious employment and calling on the psychosocial health and work well-being of young and older workers in the care sector: a longitudinal study

**DOI:** 10.1007/s00420-023-02017-z

**Published:** 2023-10-16

**Authors:** Marja Hult, Hanna Kallio, Mari Kangasniemi, Tanja Pesonen, Juho Kopra

**Affiliations:** 1 Department of Nursing Science, University of Eastern Finland, Kuopio, Finland; 2 Department of Nursing Science, University of Turku, Turku, Finland; 3 School of Computing, University of Eastern Finland, Kuopio, Finland

**Keywords:** Calling, Care work, Longitudinal, Precarious employment, Psychosocial health, Work well-being

## Abstract

**Objective:**

Employment conditions in the care sector are changing, and precarious employment (PE) is becoming more widespread, manifesting as undervaluation, adverse leadership, work overload, and inadequate control over work. This study aimed to examine changes in psychosocial health, work well-being, PE, and calling over time and explore the effects of PE and calling on psychosocial health and work well-being.

**Methods:**

The longitudinal study collected follow-up panel data in the three time points (2020, 2022, and 2023) from care workers (n = 1502), linear mixed effects models.

**Results:**

PE decreased (β =  – 0.02), and perceived work well-being increased (β = 0.04), but there were no change in psychosocial health (β =  – 0.01) and calling (β = 0.01) during the three-year period. Younger (< 39) care workers perceived higher levels of PE and had poorer psychological health. Moreover, PE had a negative effect on psychosocial health (β =  – 0.63) and work well-being (β =  – 0.68) and calling had a positive effect on psychosocial health (β = 0.41) and work well-being (β = 0.49) in multivariate models.

**Conclusion:**

PE conditions affect work performance and employee well-being and may threaten patient care; therefore, it should be further investigated in the care sector. It is noteworthy that calling still seems to be central in care work. The results deepen the understanding of the current shortage crisis in health and social care workplaces but can also provide keys to resolving the crisis.

## Introduction

Worldwide, the care sector suffers from numerous challenges. Aging populations require more care, a demand challenging to meet with an aging and declining workforce (WHO 2023). Work in the sector is physically, mentally, and emotionally burdensome, and in many countries, workers perceive the compensation offered as insufficient. The work in the care sector embodies the characteristics of precarious employment (PE), serving as an indicator of low-quality employment (Vanroelen 2019; Kreshpaj et al. 2020). PE has been conceptualized in various contexts, revealing common features such as insecure employment, inadequate income, and a lack of rights and protection (Kreshpaj et al. 2020). Care professionals constitute a demographic for whom fixed-term jobs and other forms of temporary and contractual flexibility have become increasingly prevalent (Rasmussen et al. 2019; Galbany-Estragués et al. 2022). The consequences of temporary employment can be argued to be particularly harmful in care work, impacting patient safety and potentially heightening the risk of patient mortality (Dall’Ora et al. 2020). This study addresses the growing concern of PE becoming more widespread in the female-dominated care sector (Fité-Serra et al. 2019; Hult et al. 2022). PE significantly hampers workers’ health and well-being, career development, job satisfaction, job tenure, as well as career and income development, impeding overall progress in life (Julià et al. 2017; Jonsson et al. 2019; Purkayastha et al. 2021). It also demonstrates adverse impacts on the health and well-being of care workers (Hult et al. 2022).

The most recent labor statistics in Europe highlight the substantial presence of women in the care sector, constituting one-third of the workforce and underlining its significance within the labor market (OECD 2019). Despite the prevalence of PE being notably higher among individuals with lower educational attainment (Jonsson et al. 2019), especially impacting young workers (Bodin et al. 2020), care work has not traditionally been a focal point in PE research. However, examining PE within this sector is now crucial, considering recent transformations, such as flexibilization, public sector austerity measures, the adoption of new public management practices, and increasing commercialization (Wall 2015; Fité-Serra et al. 2019; Nigenda et al. 2020). Flexible employment arrangements are primarily utilized to address staffing shortages and are a consequence of both workforce optimization and outsourcing, particularly prevalent in long-term care (Drange and Vabø 2021; Gil 2022). Furthermore, care work stands out as a profession with marked gender disparities. Women in this field face discrimination in various employment facets, including pay discrepancies and adverse working conditions (Sutela et al. 2019). It can be argued that gender constitutes a primary axis of inequality intersecting with PE (Benach et al. 2016). Despite care workers often being organized into trade unions, their bargaining power within the labor market remains typically limited (van der Cingel and Brouwer 2021).

Care professions have conventionally been characterized as a “calling” (Eley et al. 2012), defined by an intrinsic motivation, a drive to fulfil one’s life purpose through practice, and a genuine desire to help others (Emerson 2017; Shimizu et al. 2018). Being an occupation of calling and profound commitment (White 2002), care work offers a compelling context for examining the interconnectedness of PE and intrinsic job-related characteristics. Despite prevalent labor shortages, a calling to pursue a career in the care profession still attracts young individuals (Kox et al. 2020; Kallio et al. 2022). The perception of a calling has shown associations with positive outcomes, enhancing work and life satisfaction, motivation, work well-being, and finding meaning in professional life (Douglass et al. 2015; Ziedelis 2019; Kallio et al. 2022). However, being driven by a calling can also have adverse effects. For instance, it may manifest as workaholism and result in burnout, increased work-family conflicts, or the acceptance of PE situations (Hirschi et al. 2019; DePalma 2021).

To date, there is limited research evidence available on PE within the care sector. The existing scarce studies indicate that PE is manifested through temporary work, relatively low wages, a sense of vulnerability, and limited autonomy over work in this sector (Fité-Serra et al. 2019; Nigenda et al. 2020; Hult et al. 2022). The challenges posed by the COVID-19 pandemic have further exacerbated the pre-existing issues (Matilla-Santander et al. 2021), affecting job quality and well-being within the care sector (Llop-Gironés et al. 2021), especially among young workers (Moreno Martínez et al. 2022). The pandemic also extended working hours and heightened emotional burdens (Matilla-Santander et al. 2021; Purkayastha et al. 2021). Therefore, this study aims to analyse changes in PE, psychosocial health, work well-being, and the sense of calling between 2020 and 2023 among younger (≤ 39) and older (> 39) care workers. Additionally, it seeks to examine the effects of PE and the sense of calling on psychosocial health and work well-being. The study places particular emphasis on younger care workers, as they face a higher risk of entering PE (Valero et al. 2022). This research intends to offer a fresh perspective by investigating PE in care work, an area often overlooked due to the traditional perception of these professions as providing secure career paths.

## Methods

### Study design

This study utilizes a longitudinal panel design. As per Finnish legislation, this type of study does not require ethical approval. However, the Institutional Review Boards (IRBs) of the involved trade unions and the workforce-leasing company granted permission for the study. Prior to participation, participants were asked to provide informed consent through an online form.

### Participants and data collection

The initial data collection for the longitudinal panel occurred during September to November 2020 (T1). The data were collected from the members of three care workers’ trade unions and employees of a workforce-leasing company in Finland (N = 93,000). Invitation letters were sent by designated contact persons within each organization, and one trade union also informed its members about the study through its monthly newsletter. Contact persons were requested to send two additional reminders. In the first data collection round (T1), 7925 care workers enrolled in the study, yielding a response rate of 9%. After completing the questionnaire, participants were invited to provide their contact information if they wished to partake in future follow-ups. A total of 3174 participants shared their contact details, and among them, 2117 responded to the second data collection round in March 2022 (T2), representing a response rate of 67%. In February 2023 (T3), the same 3174 participants who had provided their email addresses in T1 were sent a follow-up survey, and 1806 responded (57%). The online survey tool Webropol was utilized for data collection in all three rounds. All data from these three rounds were incorporated into the study to encompass the unique experience of the COVID-19 pandemic, significantly affecting care sector workers during this period. The study participants encompassed both public and private sector workers in healthcare, social services, early education and childcare, school assistance, and youth services. The involvement of trade unions in data collection is noteworthy due to Finland’s relatively high degree of worker organization (89%) (ILO 2023), with the care sector showing a higher level of organization than the average.

### Questionnaires

Psychosocial health was assessed using the Salutogenic Health Indicator Scale (SHIS) (Bringsén et al. 2009), a validated instrument in Finnish as well (Hult and Välimäki 2023). The questionnaire comprises 12 items addressing the overall question: ‘How have you felt in the last four weeks with regard to the following?’ These items encompass experiences related to energy, morale, tension, sleep, concentration, creativity, resolution, expression of feelings, illness, energy level, social capacity, and physical function. Respondents rated these items using a semantic differential scale with six options, ranging from 6 (I have felt alert) to 1 (I have felt exhausted). The mean of all the items was calculated, where a higher mean denoted better psychosocial health. Notably, the SHIS comprehensively evaluates both mental and social dimensions of well-being, considering illness as well, recognizing it as a potential hindrance to individuals achieving their potential (Bringsén et al. 2009). Therefore, the SHIS views health as a critical resource for personal goal attainment and effective coping at work. In this study, the internal consistency of the SHIS was assessed using Cronbach’s alphas, yielding values of 0.94 in 2020, 0.94 in 2022, and 0.94 in 2023. These values align with a previous study reporting an internal consistency of α = 0.94 among healthcare workers (Ejlertsson et al. 2018).

The study also utilized the Work Experience Measurement Scale (WEMS), developed by Nilsson et al. (2013) to evaluate work well-being. This scale comprises six subscales, each addressing a specific aspect of work well-being: *Supportive working conditions* (7 items), *Internal work experiences* (6 items), *Autonomy* (4 items), *Time experience* (3 items), *Leadership* (6 items), and *Process of change* (6 items). Respondents rated each item using a six-point Likert scale, where 6 denoted “totally agree” and 1 denoted “totally disagree”. For instance, a sample item is: ‘We encourage and support each other at work.’ The mean scores for each subscale were computed, followed by the calculation of a mean for all subscales to derive a total score, where a higher total score indicated better work well-being. The internal consistency of the WEMS was assessed using Cronbach’s alphas, resulting in values of 0.94 (in 2020), 0.95 (in 2022), and 0.95 (in 2023). In previous studies involving healthcare workers, the subscale alphas have ranged between α = 0.89–0.96 (Nilsson et al. 2013; Ejlertsson et al. 2018).

Perceptions of PE were measured using the Employment Precariousness Scale (EPRES), developed by Vives et al. (2010). The EPRES includes six subscales, each rated on a scale from 0 (indicating no precariousness) to 4 (reflecting high precariousness). The subscale *Temporariness* assesses the duration of the current employment contract and the length of time working for the same employer. The *Wages* subscale measures salary level and its sufficiency in covering daily and unexpected needs. The *Disempowerment* subscale assesses the settlement of working hours and salary. The *Vulnerability* subscale includes five items: frequency of being afraid to demand better working conditions, being afraid of being fired, being treated in an authoritarian manner, being defenceless with regard to unfair treatment by superiors, and feeling easily replaceable. The *Rights* subscale includes questions about rights to parental leave, retirement, unemployment insurance, severance pay, and sickness benefits. The *Exercise of rights* subscale includes questions about the realization of the aforementioned rights in the workplace. The total score is derived as the mean of the subscale scores. Cronbach’s alphas were calculated to evaluate the internal consistency of the EPRES and resulted in values of 0.80 (in 2020), 0.78 (in 2022), and 0.77 (in 2023). The earlier studies have reported alphas of 0.83 (Vives-Vergara et al. 2017) and 0.86 (Vives et al. 2010).

The Calling and Vocation Questionnaire (CVQ) was used to measure calling (Dik et al. 2012). The full CVQ includes CVQ Search, which evaluates the search of one’s calling, and CVQ Presence, which measures a current calling. We used the CVQ Presence subscale, containing 12 items divided into three further subscales, *Transcendent summons*, *Purposeful work*, and *Prosocial orientation*, each containing four items. Response options ranged from 1 (not at all true of me) to 4 (absolutely true of me). An example item is: ‘I believe that I have been called to my current line of work.’ We calculated the mean of all items, ranging from 1 to 4, with a higher mean indicating a higher level of calling. Cronbach’s alphas for the CVQ were 0.86 (2020), 0.87 (2022), and 0.88 (2023) in this study. Dik et al. (2012) reported an alpha of 0.90. Authorized translators double translated the WEMS, EPRES, and CVQ scales and they were discussed and finalized in a research group. The questionnaire was tested with ten nurses before the data collection.

### Data analysis

The dataset had a few missing values, 2.3% at maximum; therefore, the missing data was removed listwise. The study initially involved calculating means and standard deviations (SD) for all scales used in the research, considering subscales for each of the three data collection rounds and including all respondents in each round. Subsequently, the analysis focused on data from respondents who participated in all three data collection rounds (n = 1502). To assess reliability, Cronbach’s alphas were computed for all instruments in each data collection round, with an alpha > 0.70 considered acceptable (Tavakol and Dennick 2011). Means (SD) of study variables were then compared between the two age groups (≤ 39 and > 39 years) for each study year. Intraclass correlation coefficients (ICCs) were calculated using null models for psychological health, work well-being, precarious employment (PE), and calling. In this calculation, measurements from the same individuals were treated as clusters, grouping multiple observations together. To analyze changes in psychosocial health, work well-being, PE, and calling between 2020 and 2023, linear mixed effects regression models were used, considering age (≤ 39 and > 39 years) as a covariate in the models. Finally, mixed-effects linear regressions with random slopes were employed to examine the effects of PE and calling on psychosocial health and work well-being. All statistical analyses were conducted using STATA Version 17.

## Results

Care workers’ average age was 48 years, with 21% being under 40, and they were predominantly women (92%) (Table 1). The majority of care workers had a professional degree (73%), and most of them were practical nurses (81%) working in the healthcare sector (39%). Less than a fifth (17%) had a temporary working contract.

**Table 1:** Characteristics of care workers (n = 1502)

	M (Sd)	n	%
*Age, years*	48.4 (9.9)		
≤ 39		308	20.5
> 39		1175	78.2
Work experience, in years	16.5 (11.0)		
*Gender*			
Men		94	6.3
Women		1384	92.1
*Education*			
Professional degree		1095	72.9
Bachelor’s degree		363	24.2
Master’s degree		32	2.1
*Profession*			
Practical nurse		1219	81.2
Registered nurse		134	9.0
Auxiliary staff^1^		76	5.1
Other^2^		61	4.1
*Sector*			
Healthcare		584	38.9
Social services		534	35.6
Early education and childcare		359	23.9
*Employment contract*			
Permanent		1232	82.0
Temporary		249	16.7

^1^E.g., care aides, cleaners, secretaries, ^2^E.g., kindergarten teachers and other professions, *M*  Mean, *Sd*  Standard deviation

Table 2 presents the means (SD) for the study instruments, considering all respondents in each year. The EPRES subscale of Disempowerment consistently had the lowest mean among all subscales in each data collection. Conversely, the EPRES subscale of Wages consistently had the highest mean across all data collections.

**Table 2:** Means and standard deviations (sd) of study scales at the three time points

	2020 (n = 7925)	2022 (n = 2117)	2023 (n = 1806)
	Mean (sd)	Mean (sd)	Mean (sd)
*SHIS (scale 1–6)*	3.83 (1.04)	3.84 (1.04)	3.89 (1.00)
*WEMS (scale 1–6)*	3.72 (0.86)	3.88 (0.90)	3.96 (0.85)
Supportive working conditions	3.86 (0.73)	4.21 (0.97)	4.26 (0.93)
Internal work experiences	4.56 (0.98)	4.64 (0.97)	4.66 (0.95)
Autonomy	3.42 (1.20)	3.57 (1.21)	3.72 (1.18)
Time experience	3.61 (1.30)	3.71 (1.34)	3.88 (1.28)
Leadership	3.78 (1.26)	3.93 (1.26)	4.06 (1.21)
Process of change	3.09 (1.28)	3.20 (1.31)	3.19 (1.27)
*EPRES (scale 0–4)*	1.12 (0.49)	1.05 (0.47)	1.04 (0.48)
Temporariness	0.53 (0.79)	0.48 (0.79)	0.53 (0.82)
Wages	1.77 (0.80)	1.71 (0.81)	1.71 (0.85)
Disempowerment	0.38 (0.73)	0.38 (0.73)	0.43 (0.74)
Vulnerability	1.44 (0.92)	1.25 (0.89)	1.20 (0.85)
Rights	1.38 (1.11)	1.26 (1.03)	1.21 (1.00)
Exercise of rights	1.24 (0.82)	1.18 (0.82)	1.11 (0.80)
*CVQ (scale 1–4)*	2.65 (0.57)	2.72 (0.59)	2.72 (0.59)
Transcendent summons	2.44 (0.58)	2.56 (0.62)	2.53 (0.60)
Purposeful work	2.50 (0.77)	2.54 (0.64)	2.54 (0.78)
Prosocial orientation	3.01 (0.65)	3.06 (0.59)	3.07 (0.66)

*SHIS*  Salutogenic Health Indicator Scale, *WEMS*  Work Experience Measurement Scale, *EPRES*  Employment Precariousness Scale, *CVQ*  Calling and Vocation Questionnaire

The use of multilevel models was justified, because 59% of the total variance of the SHIS (ICC = 0.589), 63% of the total variance of the WEMS (ICC = 0.625), 66% of the EPRES (ICC = 0.658), and 71% of the CVQ (ICC = 0.705) were explained by clusters (i.e., the repeated measurements of each individual were strongly correlated). The likelihood ratio test indicated that the use of both random intercept and random slope were needed (χ^2^(3) = 2017.47, p < 0.001). In the analysis using linear mixed effects models with random slopes (Table 3), no significant change in psychosocial health over time was observed. However, care workers aged over 39 exhibited slightly better (5%) psychosocial health compared to their younger counterparts. Work well-being showed a significant, albeit modest, increase over time (β = 0.037), with older care workers perceiving higher levels of work well-being than younger workers. Moreover, the perceived amount of PE decreased significantly, however, very little (β =  – 0.022), and younger care workers (39 years or less) perceiving moderately higher levels of PE (β =  – 0.187) than their older counterparts over time. No significant changes were detected in the sense of calling over time or between the different age groups regarding the perception of calling.

**Table 3:** Linear mixed effects regression analyses for the changes over time (n = 1502)

	Psychosocial health	Work well-being	Precarious employment	Calling
	β (Se)	β (Se)	β (Se)	β (Se)
Intercept	3.767 (0.205)***	2.770 (0.168)***	1.875 (0.090)***	2.447 (0.106)***
Year	– 0.006 (0.008)	0.037 (0.007)***	– 0.022 (0.004)***	0.008 (0.004)
Age, ref > 39	0.132 (0.056)*	0.128 (0.048)**	– 0.187 (0.026)***	0.053 (0.033)

^*^p < 0.05, **p < 0.01, ***p < 0.001, *β*  Standardized coefficient, *Se*  Standard error

**Fig. 1:**
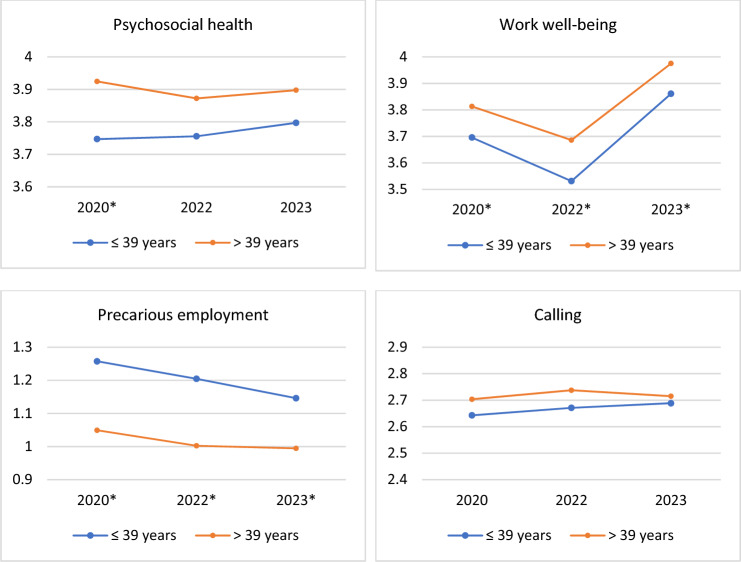
Means in study variables from 2020 to 2023, clustered by age, among care workers (n = 1502). *Statistically significant (p < 0.05) differences between the age groups

In Fig. 1, the means of the study variables and the differences between younger and older care workers are visually presented. The graphs illustrate that younger care workers experienced significantly poorer psychological health in 2020 and lower work well-being in all study years. Notably, the data indicated that PE among younger workers was 20% higher than among older workers in 2020 and 2022, with a difference of 15% in 2023. Additionally, they had slightly lower levels of calling compared to their older counterparts. However, the differences in calling were not statistically significant.

Perceived PE showed a significant negative effect (β =  – 0.679) on psychosocial health (Table 4). Conversely, the perception of calling exhibited a significant positive effect (β = 0.464) on psychosocial health. When calling was added to the model, the effects of both PE and calling slightly decreased. Regarding work well-being, PE had a significant negative effect (β =  – 0.730), while calling had a significant positive effect (β = 0.538). PE and calling reciprocally decreased each other’s effects on work well-being.

**Table 4:** Effects of precarious employment and calling on psychosocial health and work well-being among care workers (n = 1502)

	Psychosocial health^1^			Work well-being^1^		
	β (Se)	β (Se)	β (Se)	β (Se)	β (Se)	β (Se)
Intercept	5.001 (0.191)***	2.694 (0.198)***	3.900 (0.201)***	4.161 (0.151)***	1.574 (0.155)***	2.863 (0.152)***
Precarious employment	– 0.679 (0.033)***		– 0.628 (0.033)***	– 0.730 (0.026)***		– 0.676 (0.025)***
Calling		0.464 (0.028)***	0.413 (0.027)***		0.538 (0.022)***	0.487 (0.020)***

^1^Adjusted for year and age group, ***p < 0.001, *β*  Standardized coefficient, *Se*  Standard error

## Discussion

This study significantly contributes to our understanding of the well-being and work experiences of care workers, offering valuable and timely insights. Across the three-year observation period, certain trends became apparent. Notably, the psychosocial health and perceived calling of care workers remained relatively stable over time, indicating a resilient aspect of their well-being. Concurrently, a positive development was observed as perceived precarious employment (PE) among care workers decreased between 2020 and 2023. Although the decrease was very small, it is a promising sign in an often challenging and precarious field. Additionally, there was a modest increase in work well-being, showcasing a potentially improving work environment or coping mechanisms. These dynamics align with the evolving landscape of care work, which is undergoing significant transformations. The study underscores the need to recognize and address the distinctive challenges faced by care workers, particularly emphasizing the disparities between younger and older care workers. Younger workers perceive their psychosocial health and work well-being slightly lower and experience a 20% higher perception of PE compared to their older counterparts. This underlines the importance of age as a factor in understanding how individuals perceive their well-being and employment circumstances, which can have significant implications for designing targeted interventions and support systems to enhance the well-being of younger workers in the care sector.

The perceived PE levels displayed a decline during the three-year follow-up, indicating a favourable trend towards enhanced employment quality. This observation likely mirrors the challenging employment conditions brought about by the COVID-19 pandemic in 2020 (Purkayastha et al. 2021). Subsequently, as the pandemic abated, there was an observable improvement in employment circumstances. During the pandemic’s peak, the health and social care sectors implemented measures such as holiday cancellations and employee reassignments without prior consultation (Marceau et al. 2022), potentially diminishing care workers’ autonomy and rights, while amplifying feelings of uncertainty and inequity. However, it is notable that younger workers consistently reported elevated levels of PE, aligning with prior research findings (Vives et al. 2010; Bodin et al. 2020; Matilla-Santander et al. 2022).

The dimension consistently associated with the highest levels of perceived PE is inadequate compensation. This issue is particularly pronounced in Finland, where the average salary of nurses falls below the national average (Eurostat 2022). For example, in Estonia, despite significantly lower health expenditure compared to Finland, nurses receive salaries slightly above the average. On the contrary, in countries like Belgium, nurses enjoy salaries 1.6 times higher than the average. Intriguingly, nurses have conveyed that dissatisfaction with salary levels is not the primary cause for discontent or attrition within the industry. Instead, they emphasize that decent working conditions hold greater importance (Ring and Kaarakainen 2023). To enhance working conditions, a bottom-up planning approach and active involvement of staff are recommended, as care workers possess valuable insights to effectively address workflow-related challenges.

The three-year follow-up did not reveal a shift in the perceived psychosocial health of care workers, even considering the period encompassing the COVID-19 pandemic. Notably, numerous studies have underscored the detrimental impact of the COVID-19 pandemic on the health of care workers (Matilla-Santander et al. 2021; Llop-Gironés et al. 2021). Consequently, one might have anticipated a decline in psychosocial health. However, the stability of the measure can be elucidated by the fundamental assumptions of the measurement instrument employed. The instrument was grounded in a salutogenic approach, emphasizing positive health, with a pivotal focus on a sense of coherence (Bringsén et al. 2009; Hult and Välimäki 2023). According to the salutogenic theory, a sense of coherence is fortified when an individual perceives their life as meaningful, comprehensible, and manageable (Antonovsky 1996). Despite some studies indicating that interventions could augment a sense of coherence, particularly among working-aged individuals facing depression (Wagman et al. 2023), it is generally viewed as a relatively stable personal state of mind. The diminished psychosocial health and work well-being among younger workers likely underscore the dissatisfaction prevalent among them. Achieving a sense of meaningfulness in the sector proves challenging, given that the workload and demands may surpass the comprehension and control of care workers. The outcomes further illuminate that young nurses experience significantly higher levels of burnout than their older counterparts (Shapiro et al. 2022). Overall, young workers are especially susceptible to the adverse consequences of PE on their health and well-being (Creed et al. 2020).

PE showed a pronounced adverse impact on psychosocial health and work well-being, aligning with prior research that highlighted detrimental effects of PE on various health and work-related outcomes (Julià et al. 2017; Rönnblad et al. 2019; Hult et al. 2022). However, there is a notable paucity of studies focusing on care workers, emphasizing the urgent need for targeted attention to PE within the care sector. These compelling findings underscore the necessity for prompt action and intervention. A collaborative and inclusive dialogue involving representatives from both the employees and employers, as well as key decision-makers, is indispensable to recognize and effectively address the multifaceted concerns arising from PE. Additionally, occupational health physicians and the emerging field of workplace health promotion constitute crucial stakeholders in preventing PE within workplace settings. Enhancing the quality of employment within the care sector has the potential to cultivate commitment among young workers and attract new workers to the sector, contributing to its overall growth and sustainability.

The sense of calling remained stable among the study population during the follow-up period. This consistency is likely attributed to the inherent stability of a sense of calling within a supportive environment (Dalla Rosa et al. 2019), emphasizing the enduring ethical values deeply embedded in care work (Michaelson and Tosti-Kharas 2019). Hence, we posit that the robust value system of care workers remains resilient amidst the challenges their vocation poses. Additionally, these findings support the growing body of evidence regarding calling as a significant source of well-being and meaningfulness in contemporary care work (Kallio et al. 2022; Hult et al. 2023; McKenna et al. 2023). The intriguing interplay between calling and PE merits attention, although it remains a scarcely explored area (Hult et al. 2021). This study offers fresh insights into these critical determinants of work life within the care sector, where both phenomena appear to hold substantial significance. It provokes contemplations of what care work would entail without a sense of calling to sustain individuals through precarious working conditions. Moreover, a valuable discussion regarding potential improvements in working conditions if the foundation of care professions were not based on a sense of calling, would be worthwhile.

In the discourse surrounding employment quality, which categorises jobs from highly precarious to high-quality employment standards (Van Aerden et al. 2013), care work presents a paradox, embodying elements from both extremes. While efforts are ongoing to standardise PE’s definition, measurement, and reporting in public health research, we advocate for customising measurement instruments to specific contexts and professions (Vanroelen et al. 2021), particularly within the care sector work. Moreover, it has been asserted that the expansion of care work represents a significant factor contributing to job polarisation, leading to an escalation of PE (Dwyer 2013). The rising affluence and the increasing participation of highly educated women in the workforce have precipitated outsourcing services traditionally carried out within households, such as childcare and elderly care. This service demand has given rise to a sizable segment of low-skilled and low-wage care work, i.e., PE.

### Limitations

Several limitations should be acknowledged in this study. While the applied instruments are widely used and validated, some participants might have had difficulty comprehending the questions, potentially introducing bias into the results. Additionally, there could be a bias towards more active participation from care workers who perceive good health, well-being, and fair employment conditions. The initial response rate of 9% raises concerns about the representativeness of the sample. To assess potential bias, we conducted an analysis comparing the background characteristics of the first and last respondents, assuming the latter might resemble the non-respondents (Rönmark et al. 2009). However, no significant differences were observed between these groups. Furthermore, the study sample predominantly comprised workers over 40 years old (79%), indicating an underrepresentation of young care workers, constituting only 21% of the sample. This underrepresentation of young care workers may limit the generalizability of the findings to this demographic.

Moreover, during the second data collection round in 2022, rigorous collective bargaining between the representatives of nursing staff, involving two trade unions and public sector employers, started. Consequently, by the close of 2022, nearly 1,000 nurses had formally requested the removal of their professional rights from the supervisory authority (Cubelo 2023). It can be viewed that these negotiations were ultimately resolved in the nurses’ favour at the beginning of 2023, coinciding with the third data collection round. Therefore, nurses can anticipate a wage increase in the upcoming years. Knowing how these circumstances may have influenced workers’ responses to our survey during the research period is paramount.

A major strength of this study lies in its longitudinal design, allowing for the exploration of changes in psychosocial health, work well-being, PE, and calling among care workers during the challenging period of the COVID-19 pandemic. This research makes a valuable and unique contribution by extending the scope of PE research within the care sector and delving into its interrelation with calling, a traditionally intrinsic aspect of the care profession. This broader perspective offers fresh insights into the complexities of employment dynamics in the care sector, especially during a time of global crisis.

## Conclusion

The findings from this study underscore the critical importance of addressing precarious employment within the care sector, with a particular focus on the vulnerability of young workers and their well-being. Accepting care workers’ initiatives and modifications to their work is essential to establishing favourable working conditions and retaining them in the sector. Despite the shifts in the operational landscape due to the COVID-19 pandemic, care workers’ sense of calling remained unchanged. Safeguarding this sense of calling is vital for the future of care work and navigating any future crises. Investments in occupational well-being and effective management strategies are key to preserving this calling and intrinsic motivation. While younger workers may not display the same level of commitment as older generations, they still hold a solid will to meet the needs of others. Cultivating a sense of meaningfulness at work and fostering supportive leadership styles could be instrumental in retaining young workers in the care profession. Given the current shortage crisis, a thorough examination of migration patterns and individuals transitioning to other occupations is also imperative. Ensuring decent employment conditions, including fair pay, in the care sector is extremely important. In this task, future research should carefully inspect decent work indicators in the care work.
